# Automated evaluation of retinal hyperreflective foci changes in diabetic macular edema patients before and after intravitreal injection

**DOI:** 10.3389/fmed.2023.1280714

**Published:** 2023-10-06

**Authors:** Xingguo Wang, Yanyan Zhang, Yuhui Ma, William Robert Kwapong, Jianing Ying, Jiayi Lu, Shaodong Ma, Qifeng Yan, Quanyong Yi, Yitian Zhao

**Affiliations:** ^1^Cixi Biomedical Research Institute, Wenzhou Medical University, Ningbo, China; ^2^Institute of Biomedical Engineering, Ningbo Institute of Materials Technology and Engineering, Chinese Academy of Sciences, Ningbo, China; ^3^The Affiliated Ningbo Eye Hospital of Wenzhou Medical University, Ningbo, China; ^4^Department of Neurology, West China Hospital, Sichuan University, Chengdu, China; ^5^Health Science Center, Ningbo University, Ningbo, China

**Keywords:** diabetic macular edema, hyperreflective foci, optical coherence tomography, artificial intelligence, deep learning

## Abstract

**Purpose:**

Fast and automated reconstruction of retinal hyperreflective foci (HRF) is of great importance for many eye-related disease understanding. In this paper, we introduced a new automated framework, driven by recent advances in deep learning to automatically extract 12 three-dimensional parameters from the segmented hyperreflective foci in optical coherence tomography (OCT).

**Methods:**

Unlike traditional convolutional neural networks, which struggle with long-range feature correlations, we introduce a spatial and channel attention module within the bottleneck layer, integrated into the nnU-Net architecture. Spatial Attention Block aggregates features across spatial locations to capture related features, while Channel Attention Block heightens channel feature contrasts. The proposed model was trained and tested on 162 retinal OCT volumes of patients with diabetic macular edema (DME), yielding robust segmentation outcomes. We further investigate HRF’s potential as a biomarker of DME.

**Results:**

Results unveil notable discrepancies in the amount and volume of HRF subtypes. In the whole retinal layer (WR), the mean distance from HRF to the retinal pigmented epithelium was significantly reduced after treatment. In WR, the improvement in central macular thickness resulting from intravitreal injection treatment was positively correlated with the mean distance from HRF subtypes to the fovea.

**Conclusion:**

Our study demonstrates the applicability of OCT for automated quantification of retinal HRF in DME patients, offering an objective, quantitative approach for clinical and research applications.

## 1. Introduction

Diabetic retinopathy (DR) is one of the most common complications of diabetes (1). With about 1 in every 10 diabetic patients developing visual impairment due to DR (2). One of the leading causes of visual impairment in DR patients is diabetic macular edema (DME) (3). It is suggested that in DR patients, disruption of the blood-retina barrier leads to increased fluid leakage within the retina, resulting in the development of DME (4) ultimately resulting in visual loss.

In recent decades, advances in high-resolution fundus imaging techniques have led to the discovery of specific imaging features of retinal diseases, which may serve as diagnostic, predictive, and prognostic biomarkers for this disease (5). Optical coherence tomography (OCT) is an imaging tool that can help in the visualization of the intra-retinal layers. Due to its non-invasiveness, affordability and high resolution, this imaging tool is suggested as the gold standard for the diagnosis and monitoring of DME (6). ‘Hyperreflective foci’ (HRF) is a term denoting any hyperreflective lesion, focal or dotted appearance, seen at any retinal layer on OCT images (7). Reports suggest that HRF is associated with lipid extravasation (7), microglia cells (8), migrating retinal pigment epithelium (RPE) cells (9), degenerated photoreceptor cells, and visual prognosis (10), increasing its clinical significance. In the last decade, it was shown that the presence of HRF was associated with DME, and several more recent studies have indicated that HRF could serve as a promising biomarker for investigating DME, due to its association with the soluble cluster of differentiation 14 (CD14) pro-inflammatory cytokine expressed by glial cells, monocytes, and macrophages (8, 11).

However, manual annotation of HRF in OCT is time-consuming, and sometimes excessively subjective. With the rapid development of computer science, there is great potential for automatic segmentation and quantification of HRF in OCT images, with benefits for clinical practice. The segmentation algorithms for HRF can be categorized into two primary groups: traditional segmentation algorithms and deep learning-based segmentation methods. Traditional HRF segmentation approaches usually require manual parameter tuning and extensive prior knowledge. Okuwobi et al. (12) employed an automated grow-cut algorithm for HRF segmentation. It is difficult for traditional automated methods to perform accurate HRF segmentation due to boundary blurring and speckle noise within HRF images. Okuwobi et al. (13) introduced another component tree-based method to segment HRF by extracting the extreme regions from the connected areas. Still, the method is complicated and relies on handcrafted features. Deep learning techniques have achieved significant success in medical image segmentation. Yu et al. (14) modified GoogLeNet for HRF segmentation in DR using pixel-level predictions of small image patches. However, this method partially addresses the class imbalance issue, leading to the mis-segmentation of large blood vessels or low-contrast backgrounds as HRF. Xie et al. (15) modified 3D-UNet for HRF segmentation, introducing denoised and enhanced OCT images as a dual-channel input and dilation convolution in the final layer of the encoder to expand the receptive field. Nevertheless, this approach overlooks false positive outcomes caused by high-frequency noise in the NFL/GCL and IS/OS layers. Yao et al. (16) modified U-Net for HRF segmentation, enhancing gradient propagation by replacing ordinary convolution blocks with dual residual modules and integrating adaptive modules within the bottleneck layer to fuse local features and global dependencies. However, this network ignores the inappropriateness of employing deformable convolutions for the segmentation of HRF due to its small size and lack of shape information. Wei et al. (17) preprocessed images using Non-local means (NLM) filters and adopted a patch-based segmentation approach, employing a lightweight network for automated HRF segmentation. This network relies on the patch-based method, which further diminishes the limited semantic information inherent in HRF.

In this study, we presented a deep learning-based framework for the quantitative analysis of HRF in OCT images. Specifically, the main contributions of our article can be summarized as follows:

- We achieve excellent HRF segmentation performance by combining nnU-Net (18) adaptability with the advanced long-range feature-capturing abilities of channel and spatial attention modules.
- Using the proposed method, we extracted 12 parameters to characterize HRF morphology and distribution, showing significant differences in volume and amount among the three HRF sub-types in retinal OCT images.
- Using the extracted 12 HRF parameters, we evaluated changes in HRF before and after treatment and their correlation with central macular thickness (CMT) improvement.

## 2. Materials and methods

This is a retrospective, longitudinal study conducted at the Affiliated Ningbo Eye Hospital of Wenzhou Medical University (Ningbo, China) from November 2020 to July 2022. This study was approved by the ethics committee of the Affiliated Ningbo Eye Hospital of Wenzhou Medical University (ID: 20210327A), and informed written consent was obtained from each participant involved in our study according to the Declaration of Helsinki.

### 2.1. DME participants

Type 2 diabetes mellitus (DM) patients were recruited and diagnosed by an endocrine specialist. Demographic and clinical information from all patients such as age, gender, duration of DM, and systolic/diastolic blood pressure were recorded. All patients had an extensive ophthalmic examination, involving slit-lamp biomicroscopy, and assessment of intraocular pressure, axial length, and visual acuity. The inclusion criteria of our patients are as follows: 1. Diagnosed with type 2 DM; 2. Age > 18 years; 3. Macular edema, defined clinically and by a retinal thickness of >250 μm in the central subfield (19); 4. Could cooperate with OCT imaging. Exclusion criteria were as follows: 1. Myopia; 2. Presence of media opacities; 3. Inability to cooperate with OCT imaging.

### 2.2. OCT image acquisition

3D retinal imaging was performed using the OCT tool (Spectralis HRA + OCT; Heidelberg Engineering, Heidelberg, Germany, software version V6.16.2). This imaging equipment has a scanning protocol of 40,000 A-scans/s (20), with an axial resolution of 3.9 μm and a lateral resolution of 11.4 μm in high-speed mode. We acquired OCT images covering fovea-centered regions of 4.5 × 4.5 mm^2^, with 384 B-scans, and 6 × 6 mm^2^, with 512 B-scans. OCT images showing retinal abnormalities such as age macular degeneration (AMD), severe cataract, and glaucoma; images with signal quality less than 7; or with OCT artifacts present, were excluded. OCT data displayed in our study followed the OSCAR-IB quality criteria (21) and APOSTEL recommendation (22). Patients were excluded if their CMT did not increase after treatment with anti-vascular endothelial growth factor (anti-VEGF).

### 2.3. HRF and retinal layers segmentation

We introduced an automatic tool for HRF analysis in OCT images. A deep learning-based approach was employed for precise segmentation of HRF, boundaries of inner retina (IR) and outer retina (OR) in OCT images. The resulting segmentations are then used to calculate HRF parameters.

#### 2.3.1. Hyperreflective foci segmentation

**Figure 1:**
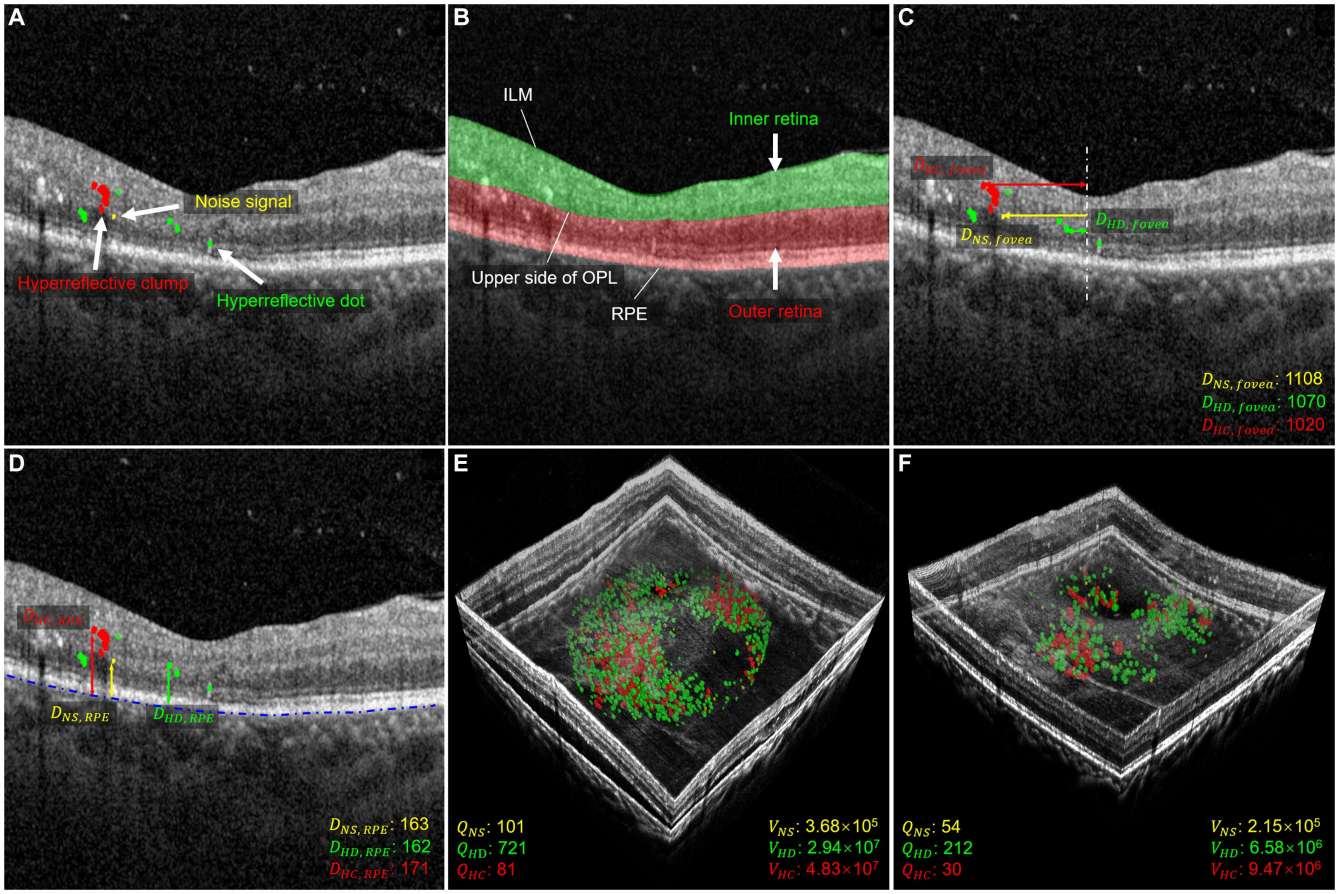
Morphology and distribution-related parameters used in quantitative measurements. **(A)** Shows the segmentation of HRF with NS in yellow, HD in green, and HC in red. **(B)** Shows the segmentation of the retina, with the inner layer in green and the outer layer in red. **(C)** Shows the distance parameter for the foveal direction of HRF. The distance between NS and fovea is shown in yellow, the distance between HD and fovea is shown in green, and the distance between HC and fovea is shown in red. The distance is measured in μm. **(D)** Shows the distance parameters between HRF and RPE. The distance between NS and RPE is shown in yellow, the distance between HD and RPE is shown in green, and the distance between HC and RPE is shown in red. The distance is measured in μm. **(E,F)** illustrate three-dimensional volume-rendered optical coherence tomography at the initial visit and six months after the initial visit, with NS in yellow, HD in green, and HC in red. For this case, the number parameters ($Q_{NS}$, $Q_{HD}$, $Q_{HC}$), and volume parameters ($V_{NS}$, $V_{HD}$, and $V_{HC}$) decreased.

HRF was defined as discrete and well-defined lesions distributed between the in-ternal limiting membrane (ILM) and retinal pigmented epithelium (RPE), with similar reflectivity to the RPE layer (8). Considering that the most HRFs cross 2–4 B-scans (15), we randomly selected 8 consecutive B-scans from each OCT volume for manual annotations of HRF. Two senior ophthalmologists made manual annotations of HRF on 140 OCT volumes, and their consensus was defined as the ground-truth. 112 OCT volumes were randomly selected for training: the rest were used for validation. The best-performing model during training was then used for the evaluation of HRF segmentation in intact OCT data from all participants, across 22 OCT volumes from 11 eyes and a total of 9,216 B-scans. Figure 1A shows the automated segmentation results indicating HRF. Section 2.4 gives a detailed description of the proposed approach.

#### 2.3.2. Inner and outer retinal layers segmentation

The distribution of HRF in the IR and OR, and their downward shift, have been previously studied (23). The IR region is defined as the region between the upper boundary of the ILM and the upper boundary of the outer plexiform layer (OPL), while the OR region is defined as the region between the upper boundary of the OPL and the lower boundary of the RPE (24). The whole retinal layer (WR) region is then defined as including both IR and OR. When HRF cross the upper boundary of OPL, they are considered located in the OR region. The IR and OR boundaries of 1,120 OCT images randomly selected from the training and validation dataset in section 2.3.1 were manually annotated by a senior ophthalmologist (Y.Y.Z). We used 896 images for training and the rest for validation. The evaluation dataset is also the same as in section 2.3.1. Figure 1B illustrates an example of IR and OR segmentation in OCT images.

### 2.4. Methods

#### 2.4.1. Network architecture

In this research, we modified the nnU-Net, to, respectively, perform two segmentation tasks: hyperreflective foci segmentation and retinal layer segmentation. The framework comprises a basic U-Net architecture library that includes 2D and 3D version.

**Figure 2:**
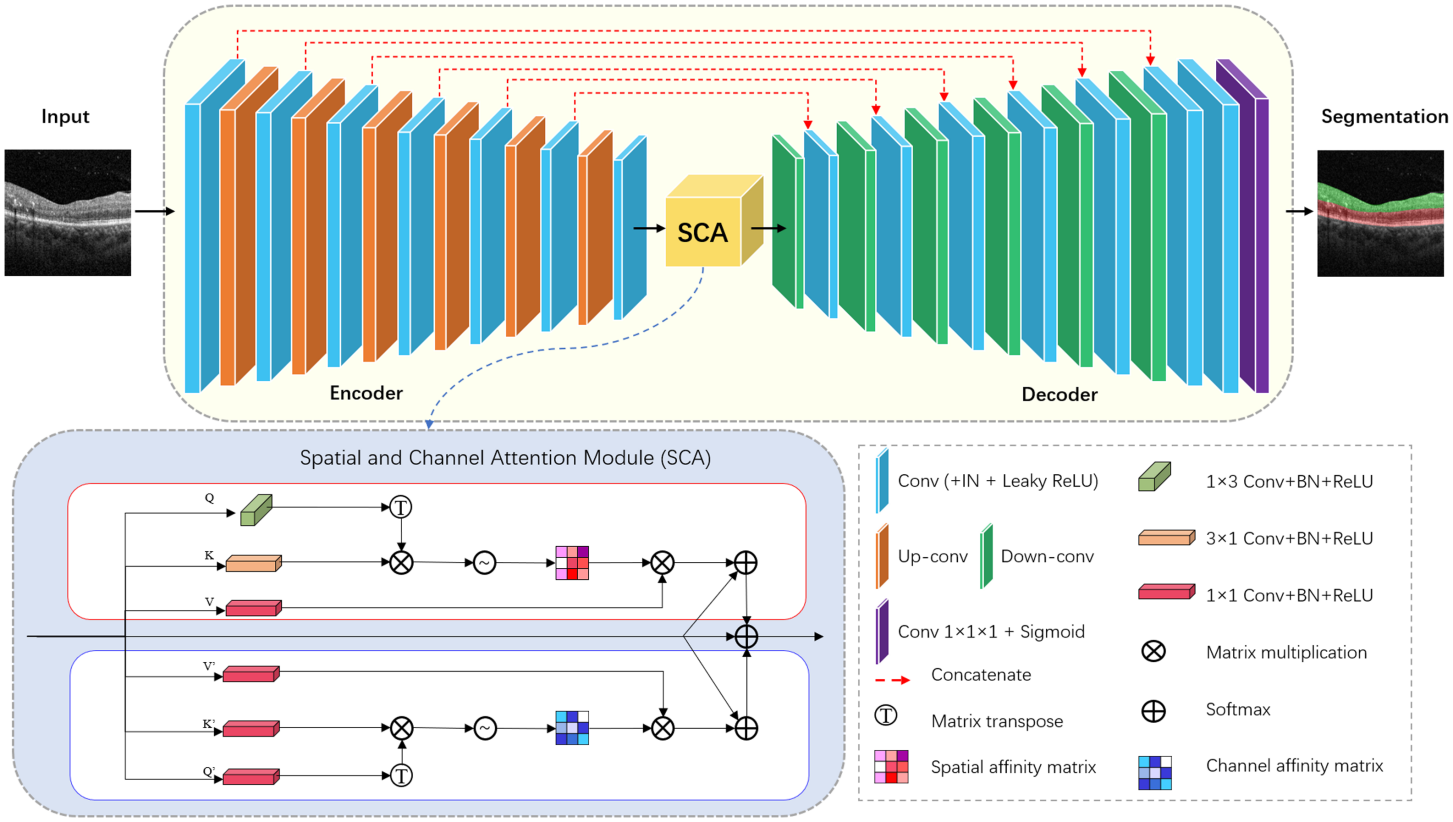
Architecture of modified nnU-Net (2D version).

For the retinal layer segmentation task, we modified the 2D version of nnU-Net as the underlying network topology. The network architecture is shown in Figure 2. The network consists of six symmetric encoder-decoder layers with skip connections, which provides detailed features from the encoder to the decoder. A 384 × 384 patch with 3 channels is first input to one 3 × 3 convolution with stride 1 to obtain the low-level feature map with 32 channels. In the encoder, each layer contains two 3 × 3 convolutions with stride 1 followed by one 3 × 3 down-convolution with stride 2. In the decoder, each layer contains a 2 × 2 up-convolution with stride 2 followed by two 3 × 3 convolutions with stride 1. Finally, the feature map of the last decoder layer is fed into one 1 × 1 convolution with stride 1 to output the segmentation map.

Convolutional neural networks with U-Net structure have higher inductive bias, but lack the ability to capture long-distance dependent features. Inspired by CS2-Net (25), we embed a spatial and channel attention (SCA) module integrating channel attention and spatial attention mechanisms at the bottleneck layer. Specifically, the features output by the encoder are fed into two sub-modules of SCA in parallel. Spatial Attention Block (SAB) aggregates features at each spatial location to correlate similar features, while Channel Attention Block (CAB) enhances the contrast of each channel feature. The spatial attention matrix models the spatial relationship between pixel features. The acquisition of intra-class spatial association can be expressed as follows:


(1) $$S_{\left(x,y\right)}=\exp\left(Q_{y}^{T}\cdot K_{x}\right)/\sum_{x^{\prime }=1}^{N}\exp\left(Q_{y}^{T}\cdot K_{x\prime }\right)$$

where $S_{\left(x,y\right)}$ represents the influence of the y position on the x position. N represents the number of features. T denotes matrix transposition. $Q_{y}$ and $K_{x}$ represent two new feature maps generated from input features, representing the vertical and horizontal directions of structural features. The channel attention matrix enhances similar channel features and reduces different channel features, which can be expressed as follows:


(2) $$C_{\left(x,y\right)}=\exp\left(F_{x}\cdot F_{y}^{T}\right)/\sum_{x^{\prime }=1}^{C}\exp\left(F_{x\prime }\cdot F_{y}^{T}\right)$$

where $C_{\left(x,y\right)}$ represents the association between the features of the x-channel and y-channel. C denotes the number of channels. T represents matrix transpose. $F_{x}$ and $F_{y}$ represent the original input features.

**Figure 3:**
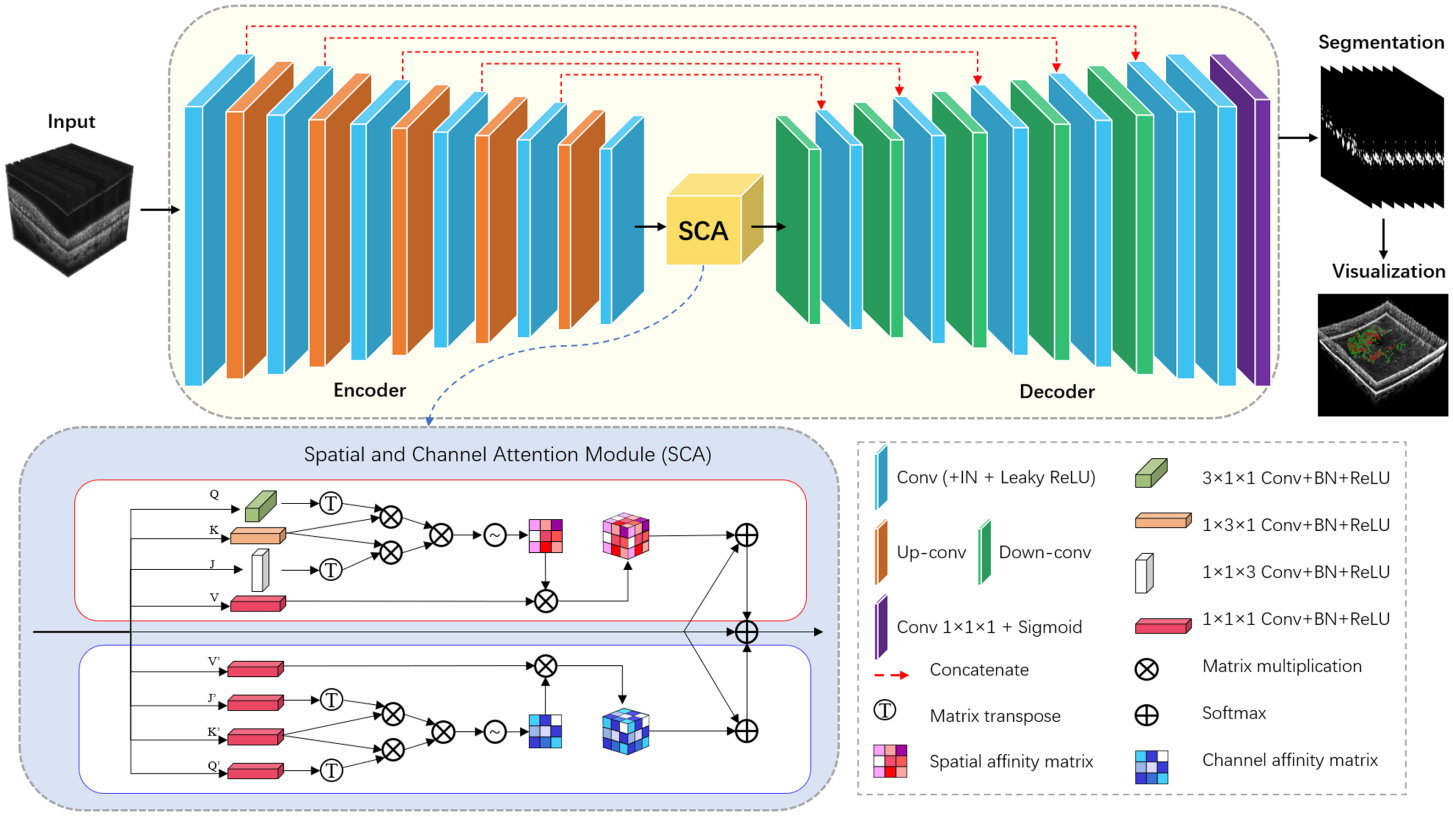
Architecture of modified nnU-Net (3D version).

For the HRF segmentation task, we have extended the modified nnU-Net from 2D to 3D. To achieve this, we have replaced all the 2D operations in both the encoder and decoder modules with 3D ones. Additionally, we have incorporated the 3D version of the SCA module into the bottleneck layer of the network. The detailed network architecture is illustrated in Figure 3.

All convolutions in the encoder and decoder adopt the form of Convolution-InstanceNorm-LeakyReLU, which are different from that in the vanilla architecture. Specifically, LeakyReLU (negative slope = 0.01) is used instead of ReLU, and instance normalization (26) is used instead of batch normalization (27). To train the network, the framework adopts a combination of dice coefficient loss and cross-entropy loss:


(3) $$L_{\text{total}}=L_{\text{dice}}+L_{CE}$$

The dice loss formula used here is a variant of that used in Drozdzal et al. (28), and it is implemented as follows:


(4) $$L_{\text{dc}}=\left(-2/|K|\right)\cdot \sum_{k\in K}\left[\sum_{i\in I}u_{i}^{k}v_{i}^{k}/\left(\sum_{i\in I}u_{i}^{k}+\sum_{i\in I}v_{i}^{k}\right)\right]$$

where u ∈ R^I × K^ denotes the softmax output of the network, v ∈ R^I × K^ denotes the one-hot encoding of the ground truth, I represent the number of pixels in a training batch and K represents the number of categories.

### 2.5. Definitions of quantitative parameters

In this study, we analyzed changes in HRF’s morphology and distribution in OCT images before and after IVI treatment. A previous study limited the maximum diameter range of HRF to 20–50 μm, which excludes the other two signals (8), refers to HRF <20 μm and > 50 μm, respectively. These signals were considered small noise signals (NS) in OCT images, and as hyperreflective clumps (HC) that appear as hard exudates in fundus images, respectively. By contrast, our study included all three types of these HRFs, allowing us to comprehensively investigate their differences in terms of number, volume, and spatial distribution. To this end, we first divided HRF into three types: NS, hyperreflective dots (HD), and HC, which are, respectively, defined as simply connected regions with a diameter range 0–20 μm, 20–50 μm, and greater than 50 μm. We then focused on 12 parameters that describe the distribution and morphological characteristics of these HRF in the retinal regions to be analyzed, as depicted in Figures 1C–F. Figures 1E,F demonstrate a 3D volume reconstruction case before and after IVI treatment. Following previous studies (8, 29), we selected a circular range of 3 mm in diameter, centered on the central macular region, for assessment of horizontal B-scans across the macular region. This region was used for analysis to ensure consistency in the region of interest across all participants.

#### 2.5.1. Morphology-related parameters

- *Noise Signal Quantity (*$Q_{NS}$*)*: Number of NS within the analyzed region.
- *Hyperreflective Dots Quantity (*$Q_{HD}$*)*: Number of HD within the analyzed region.
- *Hyperreflective Clumps Quantity (*$Q_{HC}$*)*: Number of HC within the analyzed region.
- *Noise Signal Volume (*$V_{NS}$*)*: Volume of NS within the analyzed region in μm^3^.
- *Hyperreflective Dots Volume (*$V_{HD}$*)*: Volume of HD within the analyzed region in μm^3^.
- *Hyperreflective Clumps Volume (*$V_{HC}$*)*: Volume of HC within the analyzed region in μm^3^.

#### 2.5.2. Distribution-related parameters

- *Distance between noise signal and fovea (*$D_{NS,\text{fovea}}$*)*: Distance between NS and fovea, indicating average distance of NS pixels from the foveal center in μm.
- *Distance between hyperreflective dots and fovea (*$D_{HD,\text{fovea}}$*)*: Distance between HD and fovea, indicating average distance of HD pixels from the foveal center in μm.
- *Distance between hyperreflective clumps and fovea (*$D_{HC,\text{fovea}}$*)*: Distance between HC and fovea, indicating average distance of HC pixels from the foveal center in μm.
- *Distance between noise signal and RPE (*$D_{NS,RPE}$*)*: Distance between NS and RPE, indicating average distance of NS pixels from RPE in μm.
- *Distance between hyperreflective dots and RPE (*$D_{HD,RPE}$*)*: Distance between HD and RPE, indicating average distance of HD pixels from RPE in μm.
- *Distance between hyperreflective clumps and RPE (*$D_{HC,RPE}$*)*: Distance between HC and RPE, indicating average distance of HC pixels from RPE in μm.

### 2.6. Statistical analysis

All statistical analysis was performed using version 18.0 of SPSS software (SPSS, Inc., Chicago, IL, USA). Continuous variables were expressed as mean ± standard deviation (SD) for normal data; and median and interquartile ranges (IQR) for skewed data. Categorical variables were presented as frequencies. To compare the differences among different subtypes of HRF and the differences in HRF parameters before and after treatment, the Wilcoxon signed-rank test was used, and the results were expressed as the median (IQR). To investigate the correlation between the improvement in CMT and given parameters of HRF, Spearman’s rank correlation coefficients were calculated using a non-parametric test for linear correlation. A significance level of *p* < 0.05 (two-sided test) was adopted to express statistical significance.

## 3. Results

### 3.1. Experimental results

#### 3.1.1. Implementation details

The proposed model was implemented in PyTorch using an NVIDIA GeForce 3,090 GPU with 24GB memory. The training process involved 500 epochs, and employed the following settings: Adam optimization, with an initial learning rate of 0.01; a batch size of 2 for HRF segmentation; and a batch size of 1 for retinal layer segmentation. To enhance training stability, we adopted a poly learning rate policy, with a momentum of 0.9.

#### 3.1.2. Evaluation metrics

To quantitatively assess the proposed network’s segmentation performance, we employ the following metrics. The Dice Similarity Coefficient (DSC) quantifies the agreement between HRF manually annotated by expert ophthalmologists and those automatically segmented by the proposed network, which can be defined as:


(5) $$DSC=\frac{2TP}{FP+FN+2TP}$$

We also assess our method using Intersection over Union (IOU), precision, recall, and F1-Score, defined as:


(6) $$IOU=\frac{TP}{FP+FN+TP}$$

(7) $$\text{Precision}=\frac{TP}{FP+TP}$$

(8) $$\text{Recall}=\frac{TP}{FN+TP}$$

(9) $$F1\;Sc\text{ore}=\frac{2\times \text{Precision}\times \text{Recall}}{\text{Precision}+\text{Recall}}$$

where TP indicates true positives, FP indicates false positives, TN indicates true negatives, and FN indicates false negatives.

#### 3.1.3. Comparison of different segmentation methods

In order to evaluate the effectiveness of the proposed network, we selected several state-of-the-art neural networks for comparison, including FCN (30), U-Net (31), U-Net++ (32), Res U-Net (33), 3D U-Net (34), SW-3DUNet (15), SANet (16), DBR-Net (17). The evaluation metrics utilized include the DSC, IOU, precision, recall, and F1 Score, as detailed in Table 1. We show that the proposed network outperforms other methods regarding DSC, IOU, and precision. Although the proposed method has a slightly lower recall rate than U-Net, when we consider both precision and recall comprehensively, the proposed method outperforms in terms of the F1 Score.

**Table 1:** Comparison of different segmentation methods.

Method	DSC (%)	IOU (%)	Recall (%)	Precision (%)	F1 Score (%)
FCN	59.31 ± 9.30	44.60 ± 9.32	66.07 ± 7.93	57.69 ± 9.69	59.31 ± 9.30
U-Net	62.12 ± 8.91	47.84 ± 9.42	**68.89 ± 8.44**	60.44 ± 8.64	62.12 ± 8.91
U-Net++	61.60 ± 9.26	47.48 ± 9.73	67.43 ± 6.60	60.37 ± 11.26	61.60 ± 9.26
Res U-Net	63.93 ± 8.82	50.32 ± 9.31	62.71 ± 9.20	71.37 ± 7.66	63.93 ± 8.82
3D U-Net	61.45 ± 8.64	49.41 ± 10.18	58.64 ± 12.91	65.58 ± 8.73	61.45 ± 8.64
SW-3DUNet	51.18 ± 9.87	36.26 ± 9.08	60.68 ± 12.10	47.62 ± 10.17	51.18 ± 9.87
SANet	64.33 ± 8.68	50.59 ± 9.31	63.09 ± 8.23	70.90 ± 8.40	64.33 ± 8.68
DBR-Net	51.14 ± 7.57	36.71 ± 6.77	48.88 ± 7.69	59.99 ± 6.03	51.14 ± 7.57
Proposed method	**66.83 ± 9.06**	**56.33 ± 7.08**	61.12 ± 11.9	**82.31 ± 6.39**	**66.83 ± 9.06**

^1^The variable was expressed as the mean ± standard deviation (SD). The bold value represents the optimal result for the column of indicators.

**Figure 4:**
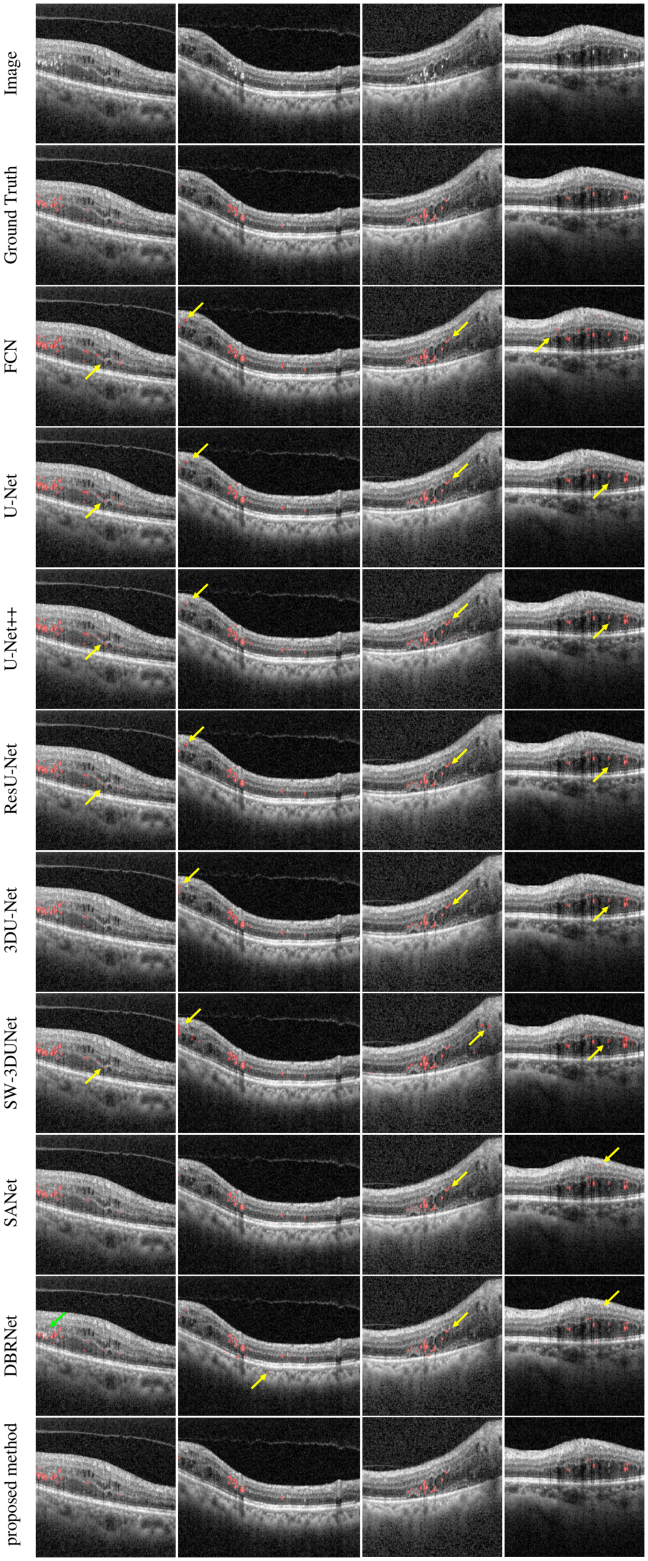
Comparison between the proposed SW-3DUNet and other methods. Yellow and green arrows represent the regions of over-segmentation and under-segmentation. B-scans in the second column are taken from fovea-centered regions of 6 × 6 mm^2^, while B-scans in the other columns are taken from fovea-centered regions of 4.5 × 4.5 mm^2^.

As seen in Figure 4, our proposed network outperforms at identifying complete HRF regions and avoiding errors in segmentation when compared to other methods in the task of HRF segmentation for DME diseases. This indicates that the proposed network can effectively extract detailed HRF features and analyze them, by combining robust pre-processing capabilities from the baseline network and embedding spatial and channel attention modules. As a result, there is a notable improvement in segmentation effectiveness.

#### 3.1.4. Ablation experiment

To demonstrate the effectiveness of the channel attention and spatial attention modules, we compared our proposed method with the baseline method and two variants.

- Baseline + SAB: We removed the CAB from this variant to assess its contribution.
- Baseline + CAB: We removed the SAB from this variant to assess its contribution.
- Baseline: We removed both SAB and CAB to evaluate their combined contribution.

Table 2 presents the experimental results for our proposed method, the baseline, and its two variants. Compared to the results of our proposed method, the variant without SAB exhibited reductions of 0.31% in DSC, 4.29% in IOU, 0.7% in recall, 0.4% in precision, and 0.31% in F1 Score. The variant without CAB showed reductions of 1.15% in DSC, 5% in IOU, 0.55% in recall, 0.75% in precision, and 1.55% in F1 Score. Removing both SAB and CAB resulted in reductions of 1.7% in DSC, 5.65% in IOU, and 2.91% in recall. Although there was a slight increase of 0.41% in precision, there was a decrease of 1.7% in F1 Score. The experimental results above demonstrate the rationality and effectiveness of embedding spatial and channel attention modules in the bottleneck layer of the baseline model.

**Table 2:** Ablation experiment.

Method	DSC (%)	IOU (%)	Recall (%)	Precision (%)	F1 score (%)
Baseline	65.13 ± 9.75	50.68 ± 9.89	58.31 ± 12.93	**82.72 ± 5.68**	65.13 ± 9.75
Baseline + SAB	65.68 ± 9.62	51.33 ± 9.65	59.57 ± 12.41	81.56 ± 6.19	65.68 ± 9.62
Baseline + CAB	66.52 ± 9.55	52.04 ± 10.10	60.42 ± 12.37	81.91 ± 6.26	66.52 ± 9.55
Proposed method	**66.83 ± 9.06**	**56.33 ± 7.08**	**61.12 ± 11.9**	82.31 ± 6.39	**66.83 ± 9.06**

^1^The variable was expressed as the mean ± standard deviation (SD). The bold value represents the optimal result for the column of indicators.

### 3.2. Quantitative parameter evaluation

We enrolled 47 eyes from 26 patients with DME, acquired with OCT (Spectralis HRA + OCT), and a total of 11 eyes from 8 patients were included in this study. We excluded 36 eyes from 18 patients from the analysis. One eye of one patient was excluded due to poor OCT image quality (motion artifacts on OCT images): 22 eyes of 11 patients were excluded due to lack of follow-up records; and 13 eyes from 7 patients were excluded due to no improvement in CMT after anti-VEGF or dexamethasone IVI treatment. The characteristics and clinical information of our study participants are displayed in Table 3. Two sets of OCT data were included for each eye, one at baseline, and one at follow-up, for a total of 9,472 OCT B-scans included in the study.

**Table 3:** Demographics.

Characteristics	
Number of patients	8
Number of eyes	11
Age, mean ± SD (range), years	48.4 ± 11.0 (33 to 61)
Male gender, *n* (%)	4 (50)
Type 2 diabetes, *n* (%)	8 (100)
Diabetes duration, mean (IQR), years	7.9 (5 to 10)
DR severity, *n* (%)	
Severe non-proliferative DR	10 (91)
Proliferative DR	1 (9)
Baseline BCVA, mean ± SD (range), LogMar	0.59 ± 0.17 (0.4 to 0.8)
Final BCVA, mean ± SD (range), LogMar	0.57 ± 0.16 (0.3 to 0.8)

^1^The retrospective nature of this study resulted in missing data, including the duration of diabetes for one patient, baseline BCVA for one eye, and follow-up BCVA for another eye. ^2^DR, diabetic retinopathy; IQR, interquartile range; BCVA, best-corrected visual acuity; LogMar, logarithm of the minimal angle of resolution.

#### 3.2.1. Parametric comparison of baseline hyperreflective foci

Table 4 compares 12 quantitative parameters of HRF, classified by different diameter sizes in WR at baseline. Among the morphology-related parameters, significant differences were observed between $Q_{NS}$ and $Q_{HD}$, $Q_{HD}$ and $Q_{HC}$, $Q_{NS}$ and $Q_{HC}$, $V_{NS}$ and $V_{HD}$, and $V_{NS}$ and $V_{HC}$ (all *p* = 0.003). No significant differences were found between $V_{HD}$ and $V_{HC}$ (*p* = 0.131). Among the distance-related parameters, the results showed no significant differences between the HRF classified according to their diameter size.

**Table 4:** Parametric comparisons of hyperreflective foci of different diameters.

Variable, in WR	Morphology-related parameters		Variable, in WR	Distribution-related parameters	
	Pre-IVI, *n* = 11	*P*		Pre-IVI, *n* = 11	*P*
$Q_{NS}$ $Q_{HD}$	60 (26–101) 239 (76–411)	0.003	$D_{NS,\text{fovea}}$ $D_{HD,\text{fovea}}$	991.35 (959.52–1072.49) 982.67 (921.75–1028.42)	0.062
$Q_{HD}$ $Q_{HC}$	239 (76–411) 20 (8–37)	0.003	$D_{HD,\text{fovea}}$ $D_{HC,\text{fovea}}$	982.67 (921.75–1028.42) 985.87 (909.17–1093.25)	1.0
$Q_{NS}$ $Q_{HC}$	60 (26–101) 20 (8–37)	0.003	$D_{NS,\text{fovea}}$ $D_{HC,\text{fovea}}$	991.35 (959.52–1072.49) 985.87 (909.17–1093.25)	0.286
$V_{NS}$ $V_{HD}$	2.88 × 10^5^ (1.14 × 10^5^–3.68 × 10^5^) 9.22 × 10^6^ (2.56 × 10^6^–1.30 × 10^7^)	0.003	$D_{NS,RPE}$ $D_{HD,RPE}$	216.45 (204.38–239.87) 213.52 (188.28–230.87)	0.424
$V_{HD}$ $V_{HC}$	9.22 × 10^6^ (2.56 × 10^6^–1.30 × 10^7^) 1.18 × 10^7^ (1.53 × 10^6^–2.86 × 10^7^)	0.131	$D_{HD,RPE}$ $D_{HC,RPE}$	213.52 (188.28–230.87) 213.86 (202.93–273.82)	0.110
$V_{NS}$ $V_{HC}$	2.88 × 10^5^ (1.14 × 10^5^–3.68 × 10^5^) 1.1810^7^ (1.53 × 10^6^–2.86 × 10^7^)	0.003	$D_{NS,RPE}$ $D_{HC,RPE}$	216.45 (204.38–239.87) 213.86 (202.93–273.82)	0.062

^1^The variable was expressed as the median (IQR); the *p* value was obtained by Wilcoxon Signed Rank Test. ^2^ WR, the whole retinal layer; IVI, intravitreal injection; $Q_{NS}$, noise signal quantity; $Q_{HD}$, hyperreflective dots quantity; $Q_{HC}$, hyperreflective clump quantity; $V_{NS}$ noise signal volume; e; $V_{HD}$, hyperreflective dots volume; $V_{HC}$, hyperreflective clump volume; $D_{NS,\text{fovea}}$, distance between noise signal and fovea; $D_{HD,\text{fovea}}$, distance between hyperreflective dots and fovea; $D_{HC,\text{fovea}}$, distance between hyperreflective clumps and fovea; $D_{NS,RPE}$, distance between noise signal and RPE; $D_{HD,RPE}$, distance between hyperreflective dots and RPE; $D_{HC,RPE}$, distance between hyperreflective clumps and RPE.

#### 3.2.2. Parametric comparison of follow-up hyperreflective foci

Due to the retrospective design of the study, OCT examinations were not performed at regular intervals. To avoid bias related to the duration of follow-up, only two consecutive follow-up visits with improvement in CMT were selected for each eye. The longitudinal study included 11 eyes from 8 patients, with a follow-up of 1.9 ± 1.6 months (range 1 to 6, median 1). During study period, all eyes were treated with intravitreal injections: 91% (10/11) of eyes received anti-VEGF injections and 9% (1/11) of eyes received dexamethasone injections. The number of intravitreal injections was 1.4 ± 0.7 (range 1 to 3, median 1).

We assessed whether changes in HRF were significant at two consecutive follow-up visits in the presence of improved CMT. Table 5 showed the comparison of the 12 quantitative parameters of HRF in WR, IR, and OR between the pre-IVI and post-IVI stages. In WR, $Q_{HD}$, $V_{NS}$, $V_{HD}$, $D_{NS,RPE}$, $D_{HD,RPE}$, $D_{HC,RPE}$ and CMT were significantly reduced in post-IVI compared with pre-IVI (*p* = 0.003, *p* = 0.033, *p* = 0.003, *p* = 0.026, *p* = 0.008, *p* = 0.004, *p* = 0.003, respectively). There were no significant changes in the other six quantitative parameters between the two phases. In IR, $D_{NS,RPE}$ and $D_{HD,RPE}$ were significantly reduced in post-IVI compared with pre-IVI (*p* = 0.016, *p* = 0.016, respectively). There were no significant changes in the other 10 quantitative parameters between the two phases. In OR, $Q_{HD}$, $V_{NS}$, $V_{HD}$, $D_{HD,RPE}$, $D_{HC,RPE}$ were significantly reduced in post-IVI compared to pre-IVI (*p* = 0.006, *p* = 0.047, *p* = 0.006, *p* = 0.004, *p* = 0.004, respectively). There were no significant changes in the other seven quantitative parameters between the two phases.

**Table 5:** Comparison of parameters between pre-IVI and post-IVI.

	WR			IR			OR		
	Pre-IVI, *n* = 11	Post-IVI, *n* = 11	*P*	Pre-IVI, *n* = 11	Post-IVI, *n* = 11	*P*	Pre-IVI, *n* = 11	Post-IVI, *n* = 11	*P*
$Q_{NS}$	60 (26–101)	64 (24–84)	0.247	14 (9–36)	15 (9–20)	0.241	43 (21–94)	48 (15–63)	0.533
$Q_{HD}$	239 (76–411)	210 (71–232)	0.003	33 (20–75)	23 (12–49)	0.173	216 (59–318)	168 (55–209)	0.006
$Q_{HC}$	20 (8–37)	27 (13–40)	0.213	0 (0–1)	0 (0–1)	0.317	19 (8–37)	27 (12–38)	0.213
$V_{NS}$	2.88 × 10^5^ (1.14 × 10^5^–3.68 × 10^5^)	2.47 × 10^5^ (1.05 × 10^5^–3.54 × 10^5^)	0.033	5.32 × 10^4^ (3.40 × 10^4^–1.35 × 10^5^)	5.27 × 10^4^ (3.85 × 10^4^–9.58 × 10^4^)	0.374	2.16 × 10^5^ (9.22 × 10^4^–3.45 × 10^5^)	1.94 × 10^5^ (6.43 × 10^4^–2.57 × 10^5^)	0.047
$V_{HD}$	9.22 × 10^6^ (2.56 × 10^6^–1.30 × 10^7^)	5.66 × 10^6^ (2.55 × 10^6^–8.45 × 10^6^)	0.003	9.71 × 10^5^ (5.74 × 10^5^–2.04 × 10^6^)	7.61 × 10^5^ (2.86 × 10^5^–1.32 × 10^6^)	0.155	7.67 × 10^6^ (1.84 × 10^6^–1.05 × 10^7^)	4.52 × 10^6^ (2.10 × 10^6^–7.69 × 10^6^)	0.006
$V_{HC}$	1.18 × 10^7^ (1.53 × 10^6^–2.85 × 10^7^)	9.97 × 10^6^ (4.65 × 10^6^–2.66 × 10^7^)	0.929	0 (0–1.53 × 10^5^)	0 (0–2.11 × 10^5^)	0.345	1.18 × 10^7^ (1.53 × 10^6^–2.84 × 10^7^)	9.97 × 10^6^ (4.65 × 10^6^–2.66 × 10^7^)	0.929
$D_{NS,\text{fovea}}$	991.35 (959.52–1072.49)	1042.95 (1014.85–1064.91)	0.534	1103.94 (1057.83–1212.58)	1086.73 (1030.12–1237.50)	0.534	954.27 (906.40–1023.99)	1011.02 (960.74–1060.23)	0.594
$D_{HD,\text{fovea}}$	980.67 (921.75–1028.42)	1005.93 (973.22–1066.87)	0.131	1035.34 (919.43–1184.14)	1014.14 (956.53–1106.73)	0.79	1000.76 (888.70–1024.25)	1011.21 (948.34–1083.34)	0.131
$D_{HC,\text{fovea}}$	985.87 (909.17–1093.25)	970.50 (833.29–1020.72)	0.79	0 (0–909.00)	0 (0–664.33)	0.893	9983.01 (909.17–1093.25)	970.50 (833.29–1022.10)	0.79
$D_{NS,RPE}$	216.45 (204.38–239.87)	190.71 (160.61–202.84)	0.026	293.66 (260.66–322.31)	234.73 (197.79–246.13)	0.013	191.16 (179.96–214.40)	174.93 (147.46–185.65)	0.075
$D_{HD,RPE}$	213.52 (188.28–230.87)	177.60 (162.27–200.99)	0.008	278.50 (270.14–336.15)	242.80 (210.56–249.98)	0.016	196.62 (181.72–211.72)	172.52 (149.30–192.06)	0.004
$D_{HC,RPE}$	213.86 (202.93–273.82)	177.44 (167.83–204.70)	0.004	0 (0–274.30)	0 (0–242.46)	0.686	213.28 (202.93–273.82)	177.44 (167.83–204.70)	0.004
CMT	401 (383–490)	315 (253–367)	0.003						

^1^the variable was expressed as the median (IQR); the *P* value was obtained by Wilcoxon Signed Rank Test. ^2^WR, the whole retinal layer; IR, the inner retinal layer; OR, the outer retinal layer; IVI, intravitreal injection; $Q_{NS}$, noise signal quantity; $Q_{HD}$, hyperreflective dots quantity; $Q_{HC}$, hyperreflective clump quantity; $V_{NS}$, noise signal volume; $V_{HD}$, hyperreflective dots volume; $V_{HC}$, hyperreflective clump volume; $D_{NS,\text{fovea}}$, distance between noise signal and fovea; $D_{HD,\text{fovea}}$, distance between hyperreflective dots and fovea; $D_{HC,\text{fovea}}$, distance between hyperreflective clumps and fovea; $D_{NS,RPE}$, distance between noise signal and RPE; $D_{HD,RPE}$, distance between hyperreflective dots and RPE; $D_{HC,RPE}$, distance between hyperreflective clumps and RPE; CMT, central macular thickness.

#### 3.2.3. Correlation between follow-up CMT changes and baseline hyperreflective foci

We assessed whether there was a significant correlation between improvement in CMT at two consecutive follow-up visits and baseline HRF. Table 6 shows the correlation between the 12 quantitative parameters of baseline HRF in WR, IR, OR, and the percentage of CMT improvement ($\Delta CMT\left(\%\right)$). In WR, significant positive correlations were shown between baseline $D_{NS,\text{fovea}}$, $D_{HD,\text{fovea}}$, $D_{HC,\text{fovea}}$, and $\Delta CMT\left(\%\right)$ (*p* = 0.015, *p* = 0.016, *p* < 0.001, respectively). There was no significant correlation between the other nine baseline quantitative parameters and $\Delta CMT\left(\%\right)$. In OR, significant positive correlations were shown between baseline $D_{NS,\text{fovea}}$, $D_{HD,\text{fovea}}$, $D_{HC,\text{fovea}}$, and $\Delta CMT\left(\%\right)$ (*p* = 0.006, *p* = 0.019, *p* < 0.001, respectively). There was no significant correlation between the other nine baseline quantitative parameters and $\Delta CMT\left(\%\right)$. In IR, there was no significant correlation between all 12 baseline quantitative parameters and $\Delta CMT\left(\%\right)$.

**Table 6:** Results of spearman correlation analysis.

$\Delta CMT\;\left(\%\right)$	Pre-IVI, *n* = 11					
	WR		IR		OR	
	$r_{s}$	*P*	$r_{s}$	*P*	$r_{s}$	*P*
$Q_{NS}$	0.355	0.285	0.118	0.729	0.273	0.17
$Q_{HD}$	0.509	0.110	0.150	0.629	0.464	0.151
$Q_{HC}$	0.600	0.051	−0.064	0.852	0.600	0.051
$V_{NS}$	0.355	0.285	0.091	0.790	0.300	0.370
$V_{HD}$	0.573	0.066	0.227	0.502	0.500	0.117
$V_{HC}$	0.527	0.090	−0.092	0.787	0.527	0.096
$D_{NS,\text{fovea}}$	0.709	0.015	0.182	0.593	0.764	0.006
$D_{HD,\text{fovea}}$	0.700	0.016	−0.236	0.484	0.691	0.019
$D_{HC,\text{fovea}}$	0.882	0.000	−0.035	0.919	0.882	0.000
$D_{NS,RPE}$	0.373	0.259	0.255	0.450	0.255	0.450
$D_{DF,RPE}$	0.200	0.555	0.336	0.312	0.309	0.355
$D_{HC,RPE}$	0.355	0.285	−0.185	0.586	0.355	0.285

^1^The *p* value was obtained by Spearman Rank Correlation analysis. $r_{s}$ is the rank correlation coefficient to indicate the closeness and direction of correlation between the two variables seen in a linear correlation. ^2^WR, the whole retinal layer; IR, the inner retinal layer; OR, the outer retinal layer; IVI, intravitreal injection; $Q_{NS}$, noise signal quantity; $Q_{HD}$, hyperreflective dots quantity; $Q_{HC}$, hyperreflective clump quantity; $V_{NS}$, noise signal volume; $V_{HD}$, hyperreflective dots volume; $V_{HC}$, hyperreflective clump volume; $D_{NS,\text{fovea}}$, distance between noise signal and fovea; $D_{HD,\text{fovea}}$, distance between hyperreflective dots and fovea; $D_{HC,\text{fovea}}$, distance between hyperreflective clumps and fovea; $D_{NS,RPE}$, distance between noise signal and RPE; $D_{HD,RPE}$, distance between hyperreflective dots and RPE; $D_{HC,RPE}$, distance between hyperreflective clumps and RPE; $\Delta CMT\left(\%\right)$ ratio of central macular thickness reduction to baseline central macular thickness.

## 4. Discussion

The given study aims to quantify HRF in OCT images as part of a retrospective study on patients with DME at baseline and follow-up. Previous studies relied on manual counting methods to quantify HRF, which is time-consuming and less reliable. In examining HRF as a potential biomarker, the existing body of literature has been inconsistent (23, 35–42), which may be due to variations in the OCT tool used, image quality, and manual segmentation of HRF. To address these challenges, our study employed artificial intelligence techniques for quantifying HRF, thereby overcoming some limitations of previous studies. With artificial intelligence, retinal images can be analyzed in a completely new way. We showed that the three subtypes of HRF were significantly different in volume and number on retinal OCT images, with HC pre-dominating in volume and HD in number. We also showed that the mean distance from HRF to RPE was reduced after IVI treatment compared to before IVI treatment. In addition, we showed that eyes with less HRF in the center of the macula showed greater reduction in macular edema after IVI treatment. These findings validate previous findings and suggest new insights, emphasizing the potential of deep learning as a powerful tool for analyzing baseline and follow-up HRF in DME patients.

### 4.1. Differences between baseline HRF parameters

Statistical analysis indicated significant disparities in both the number and volume parameters of baseline HRF subtypes. Our study validated HRF discrimination based on diameter range by analyzing baseline HRF morphological parameters. A previous study used 20 μm and 50 μm diameters to differentiate HRF subtypes (8), found a positive correlation between the number of HRF subtypes in the 20–50 μm range and the levels of CD14, without discussing the other two subtypes. Our study revealed significant differences among the three subtypes. Our findings corroborated previous studies showing that smaller HRFs merge into larger HRF (7), and show differential treatment responses (23). Furthermore, our study observed different responses to IVI treatment in the number and volume of the smallest HRF subtype, which may include microglia cells, whose activation decreased with treatment (43).

### 4.2. Follow-up findings

We showed the mean distance from HRF to RPE was significantly reduced after IVI treatment. By studying the distribution parameters of HRF in a longitudinal analysis of two consecutive follow-ups, our study indicated the tendency of HRF to migrate from the inner retina to the outer retina after IVI treatment; similar to our findings, Pemp et al. showed that DME uptake triggered the downward migration of HRF (44) into the outer retina. Notably, despite the lack of response to IVI treatment, the largest diameter HRF subtype exhibited a significant reduction in mean distance to the RPE in both OR and WR. This finding was consistent with Marmor’s mechanistic model of retinal fluid movement (45), which postulated fluid flowed across the retina due to intraocular pressure, choroidal osmolarity, and active fluid uptake by the RPE. The migration of partial HRF was impeded by narrow channels on the ELM, composed of zonular adhesions between Müller cells and photoreceptor inner segments. Consequently, this fraction of HRF aggregated in front of the ELM, forming the HRF isoform with the largest diameter, supporting the study conducted by Bolz et al. (7). However, no evidence was found to indicate migration of HRF toward or away from the fovea after IVI treatment, suggesting that IVI treatment did not significantly impact the distribution of HRF in the direction of the fovea. Further studies are required to confirm this conjecture.

### 4.3. Correlations between HRF parameters and CMT improvement

We also showed that improvement in CMT resulting from IVI treatment was positively correlated with the mean distance from HRF to the fovea in OR and WR, and not significantly correlated with other parameters. We explored the correlation between the therapeutic effect of IVI on foveal edema and the quantitative parameters of HRF at baseline by examining the quantitative parameters of HRF at baseline and the percentage improvement in CMT at two consecutive follow-up visits. A previous study (46) performed two-dimensional quantification of hard exudates in OCT enface images, and found that the area of hard exudates in the fovea at baseline was inversely correlated with BCVA at the 12th month. Similar to the aforementioned report, we showed that in both OR and WR, the percentage of IVI treatment-induced improvement in CMT was inversely correlated with the concentration of HRF in the fovea at baseline, but independent of other quantified parameters. Notably, the concentration of the largest-diameter HRF subtype in the fovea was inversely correlated with the reduction in CMT ($r_{s}$=0.882, *p* < 0.001), which could explain why there was no correlation between the concentration of baseline HRF in the fovea in IR and the reduction in CMT, since the convergence of smaller HRF subtypes to larger HRF subtypes mainly occurs in OR according to the discussion above. We speculate that future studies on the differential distribution of HRF aggregated in the fovea may be able to verify whether it produces some physiological changes that affect the outcome of IVI treatment.

### 4.4. Limitations

Our study has certain limitations. Firstly, all participants were Chinese, and enrolled from a single medical center: larger and more various samples would be an advantage. More multi-center studies should therefore be conducted on larger cohorts to confirm the reproducibility of analysis on these parameters of HRF. Secondly, the enrollment criteria for this study only included cases with a positive response to injection therapy, rather than including refractory cases. It would be more convincing to recruit subjects with definitive treatment and make a long-term follow-up comparison. Thirdly, the study design lacked untreated blank controls to derive reasons for changes in parameters before and after treatment, while the small sample size and high homogeneity made the study findings more indicative of a pilot.

## 5. Conclusion

We introduced a deep learning-based approach to quantify hyperreflective foci in OCT images of DME patients. Our retrospective analysis of 11 eyes using this method showed that it effectively quantified baseline and follow-up changes in hyperreflective foci by extracting relevant geometric parameters. In this study, we were able to validate certain findings reported in prior research and uncover novel insights: for instance, our investigation revealed that the concentration of HRF in the fovea region may influence the efficacy of IVI treatment. We believe that accurate quantification and follow-up of HRF in OCT images at baseline and during treatment may enable clinicians to monitor DME disease progression, assess treatment response and identify patients who may benefit from a personalized approach to treatment.

## Data availability statement

The raw data supporting the conclusions of this article will be made available by the authors, without undue reservation.

## Ethics statement

The studies involving humans were approved by the ethics committee of the Affiliated Ningbo Eye Hospital of Wenzhou Medical University (ID: 20210327A). The studies were conducted in accordance with the local legislation and institutional requirements. The participants provided their written informed consent to participate in this study.

## Author contributions

XW: Conceptualization, Methodology, Software, Writing – original draft, Writing – review & editing, Formal analysis. YaZ: Conceptualization, Formal analysis, Resources, Writing – review & editing. YM: Writing – review & editing. WK: Conceptualization, Formal analysis, Writing – review & editing. JY: Investigation, Writing – review & editing. JL: Software, Writing – review & editing. SM: Conceptualization, Writing – review & editing. QiY: Conceptualization, Validation, Writing – review & editing. QuY: Conceptualization, Resources, Writing – review & editing. YiZ: Conceptualization, Data curation, Funding acquisition, Investigation, Methodology, Resources, Software, Supervision, Visualization, Writing – original draft, Writing – review & editing.
